# Bedside ultrasound imaging to measure muscle cross-sectional area in preterm and term infants: a feasibility study

**DOI:** 10.1038/s41372-026-02730-w

**Published:** 2026-05-27

**Authors:** Stephanie Yeager, D. Taylor Hendrixson, Guilherme M. Cunha, Gregory C. Valentine, Katie M. Strobel

**Affiliations:** 1 Department of Pediatrics, Division of Neonatology, University of Washington, Seattle, WA USA; 2 Department of Pediatrics, Division of Neonatology, Children’s Hospital of Philadelphia, Philadelphia, PA USA; 3 Department of Pediatrics, Division of Infectious Disease, University of Washington, Seattle, WA USA; 4 Department of Radiology, University of Washington, Seattle, WA USA; 5 Department of Oral Health Sciences, University of Washington School of Dentistry, Seattle, WA USA; 6 Department of Mechanical Engineering, University of Washington College of Engineering, Seattle, WA USA; 7 Department of Obstetrics & Gynecology, Division of Maternal-Fetal Medicine at Baylor College of Medicine, Houston, TX USA; 8 Seattle Children’s Research Institute, Seattle Children’s Hospital, Seattle, WA USA; 9 Institute of Human Development and Disability, University of Washington, Seattle, WA USA

**Keywords:** Paediatrics, Nutrition disorders

## Abstract

**Background/Objective:**

Lean body mass is linked to improved outcomes, but infant muscle-specific tools are limited. Bedside ultrasound measurements of muscle cross-sectional area (CSA) may overcome these limitations.

**Study design:**

A prospective cohort of 60 infants at two neonatal intensive care units (NICUs) underwent serial ultrasound biceps CSA, rectus femoris CSA, and anthropometric measurements. Intraclass correlation coefficients (ICC) assessed measurement reliability. Spline mixed models examined associations between muscle CSA and birth gestational age, growth, postmenstrual age (PMA), and nutrition.

**Results:**

Intra- and inter-rater ICCs ranged from 0.92 to 0.99 and from 0.77 to 0.91. No safety events occurred. Biceps CSA correlated with birth gestational age, PMA, weight, length, head circumference, and mid-upper arm circumference (*p* ≤ 0.01). Rectus femoris CSA correlated with birth gestational age, weight, length, head circumference, and mid-thigh circumference (*p* < 0.05).

**Conclusion:**

Bedside ultrasound is feasible, safe, and reliable for muscle CSA measurement in NICU infants.

## Introduction

Body composition assessment has increasingly been used in neonatal and pediatric research to quantify fat mass and fat-free mass (FFM), as these measures have been linked to important clinical outcomes [1, 2]. Fat-free mass accretion reflects the accumulation of protein, which supports growth of muscle and organs. Fat-free mass gains have been strongly linked to improved cognitive and motor outcomes at 12 months of age and to greater brain growth in medically complex infants and children [1, 3, 4]. In contrast, higher fat mass accrual in preterm infants has been associated with an increased risk of cardiovascular disease, obesity, and metabolic syndrome later in life [2, 5]. Thus, obtaining in-hospital muscle measurements may be important for improving long-term outcomes.

Several methods are currently used in research to assess infant muscle mass, including air-displacement plethysmography (ADP), dual-energy x-ray absorptiometry (DXA), magnetic resonance imaging (MRI), and D3-creatine dilution. However, these methods have limitations. ADP and MRI cannot accommodate infants requiring thermoregulation [2, 6]. ADP requires minimal respiratory support. D3-creatine dilution requires medication administration and follow-up urine and saliva testing, which is feasible but can be challenging to time [2, 5, 7]. Alternatively, ultrasound is portable, bedside-accessible, radiation-free, and feasible for serial measurements in both stable and critically ill infants [8, 9]. Muscle ultrasound measurements, specifically of the biceps (upper arm) and rectus femoris (mid-thigh) muscles, have demonstrated high interrater reliability in pediatric and adult studies [10, 11]. To date, there has been limited research evaluating the use of ultrasound for muscle assessment in infants. Prior studies have primarily focused on muscle thickness, with at least one study comparing thickness to a reference body composition method [8, 12]. Muscle cross-sectional area (CSA) offers potential advantages over thickness measurements, as CSA is less affected by patient movement and the amount of pressure placed on the transducer [9, 11–15]. Current data on the longitudinal use of ultrasound to measure muscle CSA in infants, particularly across multiple muscle sites, remain limited.

Anthropometric trends in preterm infants have been inconsistently associated with long-term neurodevelopmental outcomes [16, 17]. This may reflect difficulties obtaining accurate length and head circumference measurements in preterm infants [18]. Furthermore, increased weight velocity during hospitalization has been linked to metabolic diseases later in life, likely secondary to fat mass gains [2, 19]. Multiple studies have shown that preterm infants accumulate more fat mass and less lean body mass than their term infant counterparts [8, 20, 21]. Incorporating a measure of FFM, such as muscle CSA, into routine neonatal intensive care unit (NICU) clinical care could complement existing growth assessments and provide additional insight into nutritional status and growth quality in high-risk infants.

In this pilot prospective observational study, we evaluated the feasibility, safety, and reliability of serial bedside ultrasound measurements of biceps and rectus femoris muscle CSA in preterm and critically ill term infants during NICU hospitalization, and examined their associations with standard anthropometric measures over time. We hypothesized that ultrasound-derived muscle CSA measurements would have no adverse safety outcomes, adequate intra- and interrater reliability, and would be positively associated with infant anthropometrics, including weight, length, and head circumference z-scores.

## Methods

This prospective observational cohort study was approved by the Seattle Children’s Hospital and University of Washington Institutional Review Boards. Informed consent was obtained from the infant’s parent prior to participation in the study. The primary outcome of this study was the biceps muscle cross-sectional area at each given postmenstrual age (PMA). Secondary outcomes were rectus femoris muscle cross-sectional area at each given PMA, as well as muscle CSA relationships to anthropometric measurements and nutritional intake.

### Study population

Preterm and term infants admitted to the level IV NICUs at the University of Washington Medical Center and Seattle Children’s Hospital were prospectively recruited for this pilot study. Eligibility criteria included a gestational age at birth of ≥24 weeks and a chronological age of <1 month at enrollment. Exclusion criteria were limited to known chromosomal anomalies affecting growth, history of extracorporeal life support, parental age <18 years, or an anticipated hospital stay of <14 days. Clinical illness or degree of respiratory support were not exclusion criteria, and infants were enrolled regardless of acuity to ensure the study population represented the full clinical spectrum of NICU patients. Because of the pilot nature of the study, power calculations were not performed, and we had a goal of recruiting *n* = 60 infants.

### Study procedures

Paternal and maternal anthropometrics were collected via a one-time Research Electronic Data Capture (REDCap) survey following consent [22]. Parents self-reported their height, pre-pregnancy weight, race, and ethnicity. Parental mid-upper arm circumference (MUAC) was measured at the time of enrollment by a study team member. For parents not present at enrollment, the study team provided a paper measuring tape and a standard set of instructions for obtaining MUAC at home [23]. Information on the mother’s pregnancy and delivery was abstracted from the infant’s medical record. Infant hospital course, standard anthropometric measurements, and nutritional data were serially monitored through the infant’s medical record. Trained NICU nurses obtained infant weights with calibrated electronic scales, length with a length board by two nurses, and head circumference with a flexible plastic or paper measuring tape. Every other week, study personal measured infant MUAC, mid-thigh circumference (MTC), abdominal circumference, and foot length with a flexible plastic body measuring tape immediately before ultrasound assessment [24, 25]. All measurements were obtained prior to discharge from the NICU. All relevant information was collected in a limited dataset within the REDCap electronic database [22].

**Fig. 1:**
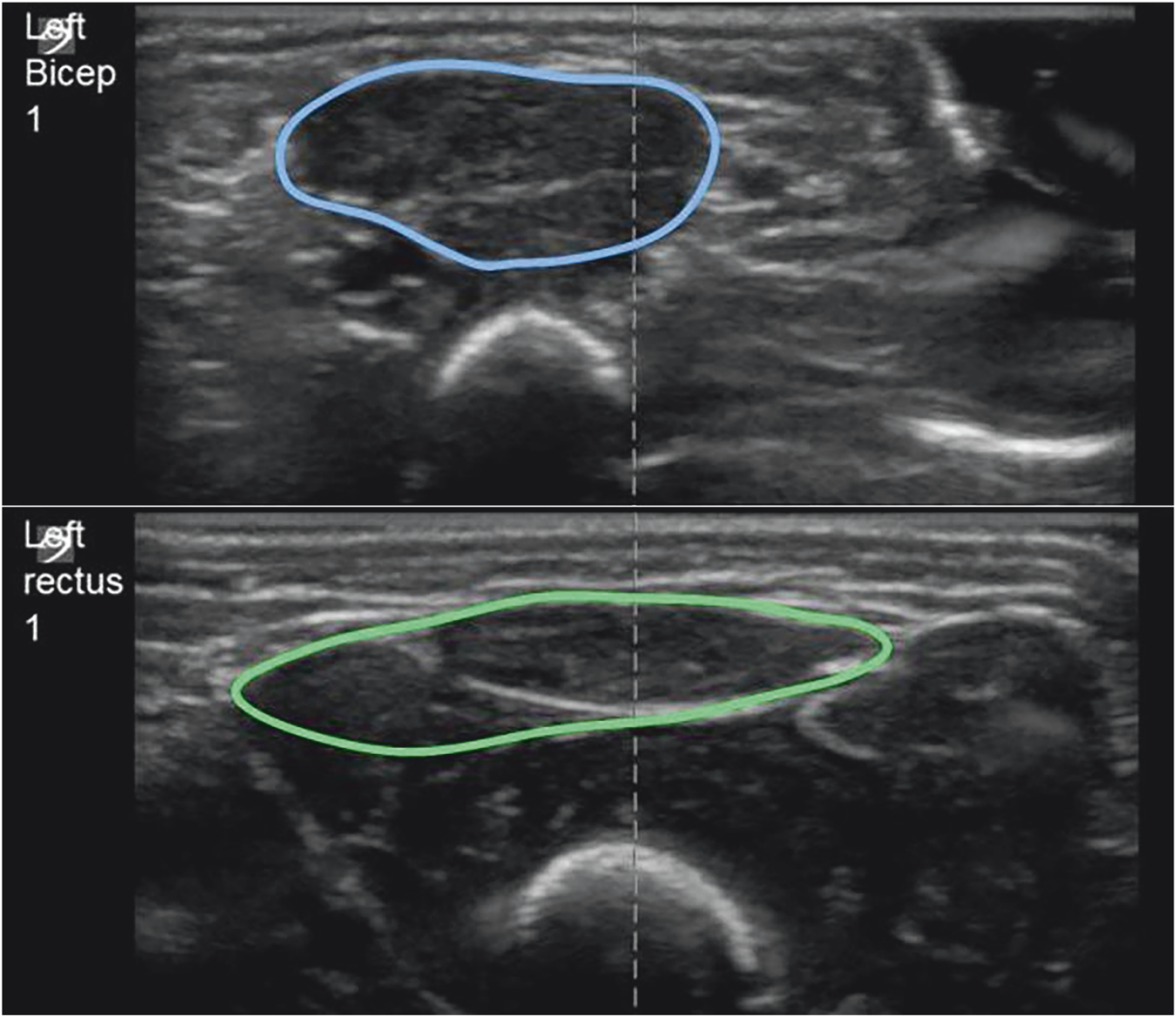
Ultrasound measurement of upper and lower extremity muscle cross-sectional area. Representative ultrasound images of the biceps (upper panel) and rectus femoris (lower panel) demonstrating muscle cross-sectional area aquisition. Images were obtained using a linear probe positioned perpendicular to the muscle belly, including the entire muscle contour and the intramuscular tendon seen as an echogenic band in the center of the muscle belly.

All ultrasound operators participated in multiple practice sessions under the supervision of an experienced musculoskeletal radiologist prior to study initiation. A standardized operating procedure, developed in collaboration with the musculoskeletal radiologist, was followed for all imaging and measurement protocols. One researcher obtained all primary ultrasound images at both sites, and two additional researchers independently obtained images for inter-rater reliability assessments. Ultrasound measurements were performed around an infant’s care time using a Sonosite XPORT or PX machine (FUJIFILM Sonosite Inc., Bothell, WA), with a 13.0-6.0 or 19.0-5.0 MHz bandwidth linear probe [26]. With the infants in a supine position, the biceps muscle was identified and marked at the mid-arm by measuring from the humeral head to the antecubital fossa with the elbow extended and the forearm in a supine position. The rectus femoris muscle was identified and marked at the mid-thigh by measuring from the femoral head to the upper pole of the patella with the subject’s leg flexed at a 45-degree angle [14, 26]. Starting at either the antecubital fossa or distal femur, the corresponding muscle tendon was identified, and the transducer moved proximally through the muscle belly until the midpoint marking was reached. Image depth was adjusted to centrally visualize the muscle and underlying bone, depending on the age and size of the subject. To ensure consistent positioning perpendicular to the muscle belly, the transducer was gently tilted until the intramuscular muscle tendon was identified as a linear echogenic structure (Fig. 1). Three images of each muscle (12 images total) were obtained in each session by the same ultrasound operator to allow for assessment of intra-rater reliability. An additional ultrasound image of each muscle was obtained independently by a second operator when assessing for inter-rater reliability. Image quality was reviewed by the ultrasound operator in real time. If the muscle border was blurred or landmarks (i.e., intramuscular muscle tendons) were not clearly visualized, a new image was captured. Additionally, images were reviewed by an ultrasound radiologist with over 15 years of experience in musculoskeletal imaging to ensure accuracy in identifying the appropriate muscle. Quantitative muscle CSA measurements were obtained using the polygon tool on Horos medical image software (horosproject.org, Annapolis, MD). The mean of the three measurements was used for analysis.

Safety was monitored throughout the study by regular review of participant medical records and direct observation during study procedures. Subjects were continuously monitored via cardiopulmonary monitors and direct visual assessment for episodes of apnea, bradycardia, or oxygen desaturations. Medical records were additionally reviewed for any documented episodes of hypothermia, hyperthermia, or unplanned extubation occurring within 1 hour of study procedures.

### Statistical analysis

**Fig. 2:**
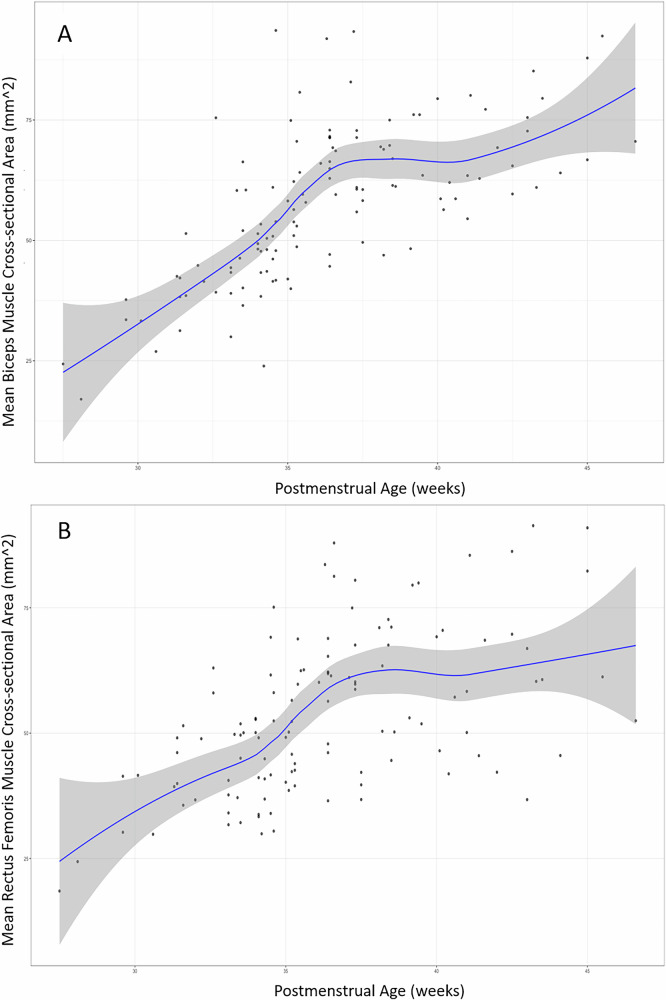
Association between muscle cross-sectional area and postmenstrual age. Locally estimated scatterplot smoothing (LOESS) curves evaluating **A** biceps and **B** rectus femoris muscle CSA and postmenstrual age in infants. We included only the first three measurements for all infants.

All statistical analysis was conducted using R statistical software (Vienna, version 4.4.2) [27]. Cohort characteristics were calculated using median and inter-quartile range (IQR) for continuous variables and frequency (%) for categorical variables. Weight, length, and head circumference z-scores were calculated using the 2013 Fenton growth curve [28]. LOESS smooth plots were generated for both biceps and rectus muscle cross-sectional area and PMA to visualize for linearity (Fig. 2). Intraclass correlation coefficients (ICC) to assess intra-rater reliability were calculated from the repeated measurements of muscle CSA obtained at each serial timepoint. For a subset of ultrasound images (*n* = 20), ICCs to assess inter-rater reliability were also calculated using the additional set of muscle CSA images obtained by a second independent measurer at the infant’s first scan.

We utilized a locally estimated scatterplot smoothing (LOESS smooth plot) to visualize for linearity. All individuals were included, but we limited each to a maximum of three measurements in the plot. Due to concerns of non-linearity, we compared a linear mixed model, a quadratic polynomial mixed model, and a natural spline mixed model utilizing differences in Akaike Information Criterion (AIC), Bayesian Information Criterion (BIC), likelihood ratio tests, and marginal versus conditional *r*^2^. In both muscle groups, the natural splines model performed the best with three degrees of freedom to allow for non-linear growth trajectories while avoiding overfitting. The spline functions describe a single smooth curve, with one component representing the overall trend and additional components allowing curvature across postmenstrual age at 35.1 weeks and 38.5 weeks. These models included a random effect for repeated measures, birth gestational age, sex, postmenstrual age, and an interaction term of birth gestational age and postmenstrual age. This modeling approach allowed for the inclusion of all available data, accommodating missing values without case-wise exclusion. Separate natural spline mixed models were then employed to examine associations between each muscle CSA measurement and anthropometric variables (weight z-score, length z-score, head circumference z-score, MUAC, MTC, abdominal circumference, and foot length), adjusting for PMA, sex, birth gestational age, birth gestational age and PMA interaction, and including subject as a random effect. Additionally, biceps and rectus femoris muscle CSA were compared to parent anthropometrics and nutritional metrics in similar models. Because ultrasound-derived muscle CSA lacks sex-based, normative z-scores, all anthropometric variables were adjusted for PMA and sex to maintain consistent comparison across measures. No adjustments for multiple comparisons were conducted, given the exploratory nature of this pilot study.

## Results

A total of 60 infants of varying gestational ages were enrolled in the study from July 2023 to August 2024. Cohort characteristics of both parents and infants are described in Table 1. Gestational ages of enrolled subjects included two extremely preterm (both 24 weeks gestational age), 17 very preterm, 17 moderate preterm, 11 late preterm, and 13 term infants. All term-corrected infants were admitted to the Seattle Children’s Level IV NICU for complex medical and surgical management. Primary reasons for term infant admissions included airway anomalies (*n* = 4), cardiac (*n* = 1), gastrointestinal (*n* = 3), neurologic (*n* = 4), and pulmonary (*n* = 1) disorders. Birth weights ranged from 720 to 4255 grams. Median birth weight z-score was −0.10 (IQR −0.62, 0.43). Mothers of the infants were healthy overall, with 13 (22%) having gestational diabetes and 8 (13%) having gestational hypertension. Of the 60 subjects enrolled, all anthropometrics, nutrition, and medical history were obtained, except for the discharge metrics for one infant who died prior to discharge and one infant with an anticipated length of stay greater than one year. Notably, the median for days to full enteral feeds (140–160 kcal/kg/day) was 7 (IQR 6, 9), and the median days to regain birth weight was 10 (IQR 7, 12). At the time of discharge, 15 infants (26%) were exclusively receiving parents’ own milk (POM), 39 (67%) were receiving a combination of POM and formula, and 4 (7%) were receiving formula exclusively. At discharge, 34 infants (59%) were fed all per mouth (PO), 16 (27%) required combination PO and nasogastric (NG) tube feeds, and 8 (14%) were exclusively fed via NG or gastrostomy tube.

**Table 1:** Description of parents (*N* = 109) and infants (*N* = 60) enrolled in the clinical study.

	Median (IQR)
Infant	
Gestational age at birth (weeks)	32.5 (30.5, 36.8)
Gestational age at discharge (weeks)	41.1 (38.2, 44.6)
Birth weight z-score	0.02 (−0.50, 0.72)
Discharge weight z-score	−0.25 (−1.17, 0.42)
Birth length z-score	−0.08 (−0.64, 0.72)
Discharge length z-score	−0.36 (−1.13, 0.83)
Birth head circumference z-score	0.43 (−0.68, 0.92)
Discharge head circumference z-score	−0.09 (−0.76, 0.09)
Maternal race	African American 8% American Indian/Native Alaskan 2% Asian 10% Native Hawaiian/Pacific Islander 7% White 76%
Maternal ethnicity	18% Hispanic 82% non-Hispanic
Paternal race	African American 8% American Indian/Native Alaskan 3% Asian 7% Native Hawaiian/Pacific Islander 3% White 72%
Paternal ethnicity	12% Hispanic 88% non-Hispanic
Maternal	
Pre-pregnancy weight (kg)	68 (59, 82)
Pre-pregnancy height (in)	64 (63, 65)
Mid-upper arm circumference (cm)	29 (27, 33)
Paternal	
Weight (kg)	93 (82, 102)
Height (in)	71 (69, 73)
Mid-upper arm circumference (cm)	35 (33, 38)
	***N (%)***
Infant	
Site, Seattle Children’s Hospital	27 (45)
Female	32 (53)
Intrauterine growth restriction	16 (27)
Necrotizing enterocolitis (Bell’s stage 2 or greater)	0 (0)
Any intracranial hemorrhage	5 (8)
Bronchopulmonary dysplasia (oxygen or positive pressure requirement at 36 weeks PMA)	5 (8)
Post-natal steroid exposure, greater than 3 doses	14 (23)
Culture positive sepsis	1 (2)
PDA requiring treatment	1 (2)
Surgery during NICU hospitalization	22 (37)
All-cause mortality	1 (2)

IQR represents the interquartile range from the 25th to the 75th percentiles. Maternal and paternal race and ethnicity were collected through a parent survey upon enrollment. Parents were able to select more than one race/ethnicity.

Study procedures, including growth measurements and bedside ultrasound imaging of muscle CSA were successfully completed in all participants every two weeks during their NICU admission. The standard time to complete all procedures was 15–20 min per session. Across the cohort, a total of 160 sets of scans were performed, plus 20 additional scans by a second operator for repeatability analysis. No adverse events were reported during the study, including temperature instability, unplanned extubation, or significant apnea, bradycardia, or desaturation events requiring intervention. Based on length of hospitalization, 37 infants underwent serial examinations approximately every two weeks, while the remaining 23 infants had a single examination prior to discharge. The average number of serial measurements across all enrolled subjects was 2.7. The intra-rater and inter-rater ICCs for each muscle are described in Table 2. The intra-rater ICCs for the four muscle CSAs ranged from 0.92 (95% confidence interval [CI] 0.84, 0.97) to 0.99 (0.98, 0.99) while the inter-rater ICCs ranged from 0.77 (0.51, 0.90) to 0.91 (0.79, 0.97).

**Table 2:** Ultrasound measurements of muscle cross-sectional area demonstrate A) intra-rater reliability and B) inter-rater reliability (ICC: intra-class correlation coefficient, CI: confidence interval).

A. Intra-rater reliability		
Measure	ICC	95% CI
Left biceps	0.99	0.98 to 0.99
Right biceps	0.98	0.95 to 0.99
Left rectus femoris	0.92	0.84 to 0.97
Right rectus femoris	0.95	0.90 to 0.98

B. Inter-rater Reliability		
Measure	ICC	95% CI
Left biceps	0.90	0.75 to 0.96
Right biceps	0.91	0.79 to 0.97
Left rectus femoris	0.87	0.70 to 0.95
Right rectus femoris	0.77	0.51 to 0.90

Ultrasound-derived biceps muscle CSA (mm²) was positively associated with PMA, birth gestational age, and the interaction of birth gestational age and postmenstrual age (*p* ≤ 0.01) in adjusted models (Supplementary Table 1). Table 3 presents natural spline models of biceps CSA with various clinical and anthropometric measures, adjusted for postmenstrual age, gestational age, and sex. With higher weight-for-age z-scores, we observed greater biceps CSA (estimate 5.7 mm^2^ [95% CI 3.8, 7.5], *p* < 0.001). Similarly, with increasing length-for-age and head circumference-for-age z-scores, biceps CSA increased (3.4 mm^2^ [95% CI 1.6, 4.7] *p* < 0.001; 1.8 mm [95% CI 0.4, 3.1], *p* = 0.01). Greater infant MUAC was also associated with increased biceps CSA (4.0 mm^2^ [95% CI 2.1, 5.9], *p* < 0.001). Longer time to full enteral feeds was associated with lower biceps CSA (−0.5 mm^2^ [95% CI −0.8, −0.2], *p* = 0.003). Parent anthropometrics and protein intake were not associated with biceps CSA.

**Table 3:** Spline mixed model of biceps and rectus femoris muscle cross-sectional area and their relationship to individual measures of interest.

Biceps CSA and measure of interest	Estimate (95% CI)	*p*-value
Infant weight z-score	5.7 (3.8, 7.5)	<0.001
Infant length z-score	3.4 (1.6, 4.7)	<0.001
Infant head circumference z-score	1.8 (0.4, 3.1)	0.01
Infant mid-upper arm circumference	4.0 (2.1, 5.9)	<0.001
Infant protein prescription	−0.9 (−3.3, 1.5)	0.5
Infant time to full feeds	−0.5 (−0.8, −0.2)	0.003
Maternal weight	−0.02 (−0.09, 0.05)	0.6
Maternal height	0.2 (−1.0, 1.5)	0.7
Maternal mid-upper arm circumference	0.2 (−0.5, 0.9)	0.6
Paternal weight	−0.02 (−0.09, 0.07)	0.8
Paternal height	−0.2 (−1.3, 0.9)	0.7
Paternal mid-upper arm circumference	0.2 (−0.7, 1.0)	0.7
**Rectus Femoris CSA and measure of interest**	**Estimate (95% CI)**	***p***-**value**
Infant weight z-score	5.1 (3.2, 7.7)	<0.001
Infant length z-score	2.2 (0.3, 4.1)	0.03
Infant head circumference z-score	1.9 (0.4, 3.4)	0.02
Infant mid-thigh circumference	3.1 (1.4, 4.8)	<0.001
Infant protein prescription	1.1 (−2.0, 4.1)	0.5
Infant time to full feeds	−0.5 (−0.8, −0.2)	0.004
Maternal weight	−0.002 (−0.08, 0.08)	0.9
Maternal height	0.5 (−1.1, 1.5)	0.8
Maternal mid-upper arm circumference	0.07 (−0.7, 0.8)	0.8
Paternal weight	−0.2 (−0.08, 0.07)	0.9
Paternal height	0.2 (−0.9, 1.3)	0.8
Paternal mid-upper arm circumference	0.1 (−0.8, 1.1)	0.9

All models adjusted for repeated measures, postmenstrual age, birth gestational age, birth gestational age*postmenstrual age, and sex.

Rectus femoris muscle CSA (mm²) was positively associated with birth gestational age but not postmenstrual age or the interaction of birth gestational age and postmenstrual age in adjusted models (Supplementary Table 1). As shown in Table 3, natural spline models of rectus femoris CSA adjusted for gestational age and sex demonstrated that rectus femoris CSA increased with increasing weight-for-age, length-for-age, and head circumference-for-age z-scores (5.1 mm^2^ [95% CI 3.2, 7.7], *p* < 0.001; 2.2 mm^2^ [95% CI 0.3, 4.1], *p* = 0.03; 1.9 mm^2^ [95% CI 0.4, 3.4], *p* = 0.02). Rectus femoris CSA was also strongly associated with MTC (3.1 mm^2^, [95% CI 1.4, 4.8], *p* < 0.001). Conversely, with increasing days to full enteral feeds, rectus femoris CSA tended to be lower (−0.5 mm^2^ [95% CI −0.8, −0.2], *p* = 0.004). No significant associations were found between rectus femoris CSA and parental anthropometrics or protein prescription.

## Discussion

This pilot study aimed to evaluate the safety, reliability, and feasibility of ultrasound measurements of muscle cross-sectional area in infants. Our findings demonstrate that bedside ultrasound reliably and successfully identified muscle anatomy for measuring the CSA of the biceps and rectus femoris. Importantly, these measurements were well-tolerated by all subjects, including critically ill infants. We observed significant links between ultrasound-derived CSA measurements and various anthropometric measures.

The present study underscores the safety and reliability of using bedside ultrasound to measure preterm and term infant muscle CSA. Despite the clinical status of our study subjects, many of whom were medically fragile and required invasive ventilation, gentle handling due to a difficult airway, supplemental oxygen, and thermoregulation via isolettes, no adverse events occurred during ultrasound procedures, supporting its safety. These findings further highlight the strengths of bedside ultrasound imaging compared with established body composition tools such as MRI or air displacement plethysmography, which have limitations for routine clinical use and are not feasible for critically ill infants. This tool may be an excellent adjunct to /D3-creatine to assess skeletal muscle mass over time in medically fragile infants [7, 29].

Our study had excellent intra- and inter-rater reliability for rectus and biceps measurements. Notably, inter-rater reliability was higher for the biceps than for the rectus femoris, though this trend was not statistically significant. Although this may partly reflect the smaller number of reproducibility scans (20 vs. 160 total scans), we hypothesize that the difference could be related to the relative ease of stabilizing infants’ arms compared with their legs during scanning. Prior studies in neonates have reported lower reliability when using muscle thickness rather than CSA, likely due to increased susceptibility to movement, positioning, angulation, and probe pressure [9, 11, 12]. CSA measurements are less affected by these variables, and probe positioning can be confirmed by visualizing the intramuscular tendon [13, 14, 30].

We observed steady increases in biceps CSA over time, supporting its feasibility as a longitudinal nutritional metric in NICU infants. The strong associations between biceps CSA and PMA are notable, as research has highlighted the importance of and need for a method to assess postnatal lean body mass accrual in relation to growth trajectories and long-term health outcomes in infants [1, 3, 4, 6]. Biceps CSA was influenced by birth gestational age and postmenstrual age, consistent with other body composition studies [8, 31]. Notably, rectus femoris CSA did not show a significant relationship with PMA but was dependent on birth gestational age. This, along with lower reliability, may make it a weaker serial marker of body composition. However, given the limited sample size, we may have been underpowered to detect this association.

By leveraging muscle CSA as a representation of fat-free mass, our study establishes strong associations with clinically relevant growth parameters, supporting the potential of ultrasound-derived muscle CSA measurements to serve as reliable biomarkers of growth and nutritional status in infants [32–34]. To our knowledge, this is the first study to demonstrate significant associations between the CSA of the biceps and rectus femoris muscles and standard infant nutritional markers. Biceps CSA demonstrated strong positive associations with PMA, weight z-score, length z-score, and head circumference z-score. Rectus femoris CSA was significantly associated with weight z-score, length z-score, and head circumference z-score. Prior work with other body composition methods has shown similar patterns, with DXA-derived lean mass and D3-creatine dilution-derived muscle mass increasing in proportion to postnatal weight gain, and ADP-derived FFM z-scores showing moderate associations with weight growth trajectories [7, 35, 36]. The associations observed between muscle CSA, PMA, and anthropometric measures in our study align with those reported in clinically validated body composition tools.

Additionally, biceps CSA was strongly associated with mid-upper arm circumference, a validated nutritional proxy in term infants [23, 37, 38]. Rectus femoris CSA also correlated strongly with MTC. As with MUAC, MTC has been used as a proxy to assess infant growth in term infants, particularly in resource-constrained settings [31, 39, 40]. Future studies should explore MUAC and MTC trajectories and their relationship to anthropometrics in preterm infants. Both biceps and rectus femoris muscle CSA were inversely correlated with the number of days to full enteral feedings. This finding, along with work by others, suggests that minimizing early delays in advancing feedings may support muscle growth and FFM accretion [41].

We found no significant associations between infant muscle CSA and parental anthropometrics, despite growing evidence that maternal and paternal growth metrics influence neonatal body composition [42–44]. This may reflect our limited sample size, reliance on self-reported parental measurements, and the fact that all enrolled infants required intensive care, unlike many prior studies conducted in healthy newborns. These factors may have contributed to the lack of significant associations observed, but additional research is warranted to examine the associations between parental anthropometrics and infant body composition in larger cohorts of critically ill infants.

Although this study provides important pilot data on bedside ultrasound imaging as a tool for assessing infant muscle area and lean body mass, it has several limitations. The study was conducted at two centers in a single city, limiting generalizability. Although we aimed to demonstrate feasibility and safety across a heterogeneous population of preterm infants and infants with congenital anomalies, we had a limited sample of extremely preterm infants born at less than 28 weeks PMA. This, along with our limited sample size, limits our ability to examine how body composition trajectories vary by gestational age. In addition, the ultrasound measurements were not validated against an established body composition tool. Therefore, the clinical significance of muscle CSA measurements in this population remains to be established. Future studies by our research team plan to address these limitations by validating ultrasound-derived muscle CSA against gold-standard methods and in larger cohorts.

In conclusion, bedside ultrasound is a safe, reliable, and feasible tool to measure infant muscle CSA. Ultrasound is a readily available, non-invasive tool that is routinely used on both preterm and critically ill infants, making it an ideal tool for bedside fat-free mass assessment. Given the known relationship between FFM and clinical outcomes, ultrasound measurements of muscle CSA could enable earlier detection of suboptimal growth, guide nutritional optimization, and support targeted interventions to improve long-term health outcomes in critically ill infants. Future studies should replicate these findings in larger, more diverse cohorts and validate them against established tools prior to clinical implementation.

## Supplementary information

Supplementary Table 1

## Data Availability

The data generated during this study are not publicly available due to restrictions involving protected health information, but are available from the corresponding author upon reasonable request with appropriate institutional approvals.
