# Supplementary material for: Bedside ultrasound imaging to measure muscle cross-sectional area in preterm and term infants: a feasibility study
Source: J Perinatol. 2026 May 27;46(7):1216–23. doi: 10.1038/s41372-026-02730-w (PMC13423790; doi:10.1038/s41372-026-02730-w)
Supplement: Supplementary file 1 — Supplementary Table 1 [file 41372_2026_2730_MOESM1_ESM.docx]

**Supplementary Table 1.** Natural spline mixed model assessing the relationship of sex, birth gestational age (GA), postmenstrual age, and the interaction of birth gestational age and postmenstrual age on biceps and rectus femoris CSA.

| **Biceps CSA model (natural spline)** | | |
| --- | --- | --- |
| **Variable** | **Estimate (95% CI)** | **p-value** |
| Sex | -3.5 (-8.7, 2.1) | 0.2 |
| Birth gestational age | -7.9 (-10.8, -5.0) | <0.001 |
| Postmenstrual age (natural spline, 3 DF)^1^ |  | 0.01^2^ |
| Basis function 1 | -52.9 (-126.9, 21.9) | 0.2 |
| Basis function 2 | -88.8 (-254.3, 78.4) | 0.3 |
| Basis function 3 | 181.8 (-31.3, 394.5) | 0.1 |
| Postmenstrual age*birth GA (natural spline, 3 DF)^1^ |  | 0.0005^3^ |
| Postmenstrual age (function 1)*birth GA | 4.0 (1.5, 6.4) | 0.002 |
| Postmenstrual age (function 2)*birth GA | 9.7 (4.0, 15.3) | 0.001 |
| Postmenstrual age (function 3)*birth GA | -1.5 (-6.9, 4.0) | 0.6 |
| **Rectus femoris CSA model (natural spline)** | | |
| **Variable** | **Estimate (95% CI)** | **p-value** |
| Sex | -3.5 (-8.7, 2.1) | 0.2 |
| Birth gestational age | -6.4 (-10.3, -2.4) | 0.002 |
| Postmenstrual age (natural spline, 3 DF)^1^ |  | 0.3^2^ |
| Basis function 1 | 29.0 (-69.3, 130.0) | 0.6 |
| Basis function 2 | -48.9 (-271.9, 182.7) | 0.7 |
| Basis function 3 | 64.2 (-215.0, 346.8) | 0.7 |
| Postmenstrual age*birth GA (natural spline, 3 DF)^1^ |  | 0.2^3^ |
| Postmenstrual age (function 1)*birth GA | 1.4 (-1.9, 4.6) | 0.4 |
| Postmenstrual age (function 2)*birth GA | 7.1 (-0.9, 14.7) | 0.08 |
| Postmenstrual age (function 3)*birth GA | 1.7 (-5.6, 8.9) | 0.7 |

^1^ Individual basis function coefficients are not directly interpretable
^2^ Overall test for postmenstrual age effect
^3^ Overall test for postmenstrual age and birth gestational age interaction effect
